# Genetic Characterization and Population Structure of Mozambique’s Sesame (*Sesamum indicum* L.) Accessions Using DArTseq-Derived SNP Markers

**DOI:** 10.3390/genes17050528

**Published:** 2026-04-29

**Authors:** Winfred Nthamo Muteti, Rogerio Marcos Chiulele, Wilfred Abincha

**Affiliations:** 1 Department of Crop Production, Faculty of Agronomy and Forest Engineering, Eduardo Mondlane University, 3453 Avenida Julius Nyerere, Maputo P.O. Box 257, Mozambique; winfredmuteti@uem.ac.mz; 2 Centre of Excellence in Agri-Food Systems and Nutrition (CE-AFSN), Eduardo Mondlane University, 5 Andar Edificio da Reitorio, Praca 25 de Junho, Maputo P.O. Box 257, Mozambique; 3 Department of Research and Development, Kentegra Biotechnology Holdings LLC, Nairobi P.O. Box 566-00502, Kenya; wabincha@gmail.com

**Keywords:** *Sesamum indicum*, DArTseq, SNPs, population structure, genetic diversity, next-generation sequencing (NGS), genotyping-by-sequencing (GBS), Mozambique, cluster analysis, DAPC

## Abstract

Background/Objective: Sesame (*Sesamum indicum* L.) is a nutritionally and economically important oilseed crop that is grown predominantly by smallholder farmers in Mozambique. However, its breeding process is constrained by a limited understanding of the genetic diversity in sesame germplasm. Therefore, this study determined the genetic diversity and population structure of a panel of 109 sesame accessions from Instituto de Investigação Agrária de Mocambique (IIAM) using DArTseq SNPs. Methods: The generated 14,763 SNPs were filtered, retaining 11,502 high-quality SNPs for this study. Results: Overall genetic diversity was moderate (mean He = 0.30, Ho = 0.30, MAF = 0.21, PIC = 0.25). Population structure analysis using sparse non-negative matrix factorization identified eight subpopulations, consistent with principal component analysis implemented via the Latent factor mixed model. Discriminant analysis of principal components (DAPC) and Ward’s hierarchical clustering based on Nei’s distance resolved the same eight clusters, although DAPC revealed overlap among clusters, consistent with extensive admixture. Analysis of molecular variance showed that 85.85% of total molecular variation was within subpopulations and 14.15% among the subpopulations. Pairwise fixation indices (ranging from 0.02 to 0.10) identified divergent subpopulations 7 and 1 as suitable candidates for hybridization. Within subpopulations, observed heterozygosity exceeded expected heterozygosity, likely reflecting residual heterozygosity in sesame landraces, admixture, reverse Wahlund effect and scoring of paralogs as heterozygous SNPs. Conclusions: Overall, this study provided insights into sesame’s genetic diversity in Mozambique, contributing to germplasm conservation and informed parental selection.

## 1. Introduction

Sesame (*Sesamum indicum* L.) is one of the world’s oldest cultivated oilseed crops, with a diploid genome (2n = 26) and a cultivation history of more than 5000 years [1]. The crop belongs to the family *Pedaliaceae* and is grown predominantly in tropical and subtropical regions [2]. Sesame seed is valued for its high oil content (50–60%) and 18–25% protein content [3]. The unique lignans, such as sesamin, sesamolin, and sesamol, contribute to high oxidative stability and provide pharmaceutical benefits, including anti-hypertensive and anti-cancer effects [4]. Globally, sesame is a source of income for millions of smallholder farmers in arid and semi-arid regions, with global annual production exceeding six million metric tons. In recent years, sub-Saharan Africa has emerged as a major producer, accounting for approximately 60% of global sesame production [5].

In Mozambique, sesame, locally known as *Gergelim*, is an important agricultural export. It is the country’s fourth most valuable agricultural export, after sugar, pigeon peas, and tobacco [6]. According to [5], the export quantity has grown consistently over the last five years, from 58,784 tonnes in 2019 to 128,433 tonnes in 2023 [7]. Sesame is primarily grown in the northern and central regions, including Nampula, Cabo Delgado, Zambézia, Tete, and Sofala, with over 588,000 producers [6]. However, the average yield of sesame in Mozambique is relatively low (541 kg/ha) for small-scale farmers, with a potential of 1500 kg/ha [8]. The low yields can be attributed to abiotic and biotic stresses, seed recycling, poor agronomic practices, and varietal limitations [9,10]. To bridge the productivity gap, understanding and exploiting sesame’s genetic diversity is essential for improvement programs.

Genetic diversity can be assessed through morphological, biochemical, and molecular markers. While morphological markers provide preliminary information, molecular markers report environmentally independent genomic variation at higher resolution [11]. Different molecular markers have been used to assess genetic diversity in sesame, including RAPDs [12,13], SSRs [14,15], AFLPs [16,17], and SRAPs [18,19]. However, the advancement of next-generation sequencing (NGS) has revolutionized the study of sesame genetic resources, making SNPs the preferred marker choice in genetic diversity studies due to their abundance, precision, and genome-wide distribution [20].

NGS applications in sesame include: whole-genome resequencing (WGS) used to detect high-quality SNPs in Saudi and exotic germplasm [21], genotyping-by-sequencing (GBS) utilized by [22] to characterize 501 accessions in the USDA collection, and diversity array technology sequencing (DArTseq) applied to evaluate 300 Ethiopian accessions [23]. Using restriction enzymes, DArTseq digests the genome DNA and then uses high-throughput sequencing to identify markers. This method produces dominant and co-dominant markers (silicoDArT and SNPs, respectively) [24]. The DArTseq genotyping-by-sequencing platform is a widely used genotyping-by-sequencing platform due to its efficiency, low cost, and speed [25]. DArT technology has been applied to mustard [26], soybean [27], and sweet potato [28], among other crops. However, there are few studies on its application in sesame, especially in genomic studies of Mozambican sesame germplasm.

The present study was therefore designed to assess genetic diversity and the population structure of a diverse panel of sesame accessions from Mozambique using SNP markers generated by DArTseq. The findings provide a foundation for germplasm conservation and informed parental selection.

## 2. Materials and Methods

### 2.1. Plant Materials

A panel of 109 sesame accessions, comprising landraces and cultivars, was sourced from Instituto de Investigação Agrária de Mocambique (IIAM), Nampula, Mozambique, and was used in this study (see Supplementary Materials).

### 2.2. Sample Collection, DNA Extraction, and Genotyping

For genotyping, seed samples from 109 sesame accessions were sent to SEQART Africa, Nairobi, Kenya. Four seeds from each accession were planted in a seedling tray at SEQART Africa. After 15 days, fresh healthy tissue was collected from each of the four seedlings using a leaf puncher and pooled. A NucleoMag Plant DNA extraction kit was used to isolate DNA from leaf tissue at concentration ranging from 50 to 100 ng/µL. On 0.8% agarose, the amount and quality of genomic DNA were assessed. The DArTseq complexity-reduction method [29] was used to construct the libraries. The protocol involved digesting genomic DNA with a mixture of *PstI* and *MseI* enzymes, ligating barcoded and common adapters, and PCR amplifying adapter-ligated fragments. Sequencing was performed on the NovaSeq X platform as single-end reads over 138 cycles. GBS was used for quality control [30] via DArTseq™ technology. DArTseq markers (SilicoDArT and SNP) were scored using DArTsoft14, an internal marker scoring pipeline based on algorithms. The markers were aligned to the *S. indicum* genome (genome assembly: ASM2616843v1; https://www.ncbi.nlm.nih.gov/datasets/genome/GCA_026168435.1/, accessed on 28 December 2025) [31].

### 2.3. Quality Control and Genetic Diversity Analysis

Quality control of the DArTseq-generated SNPs was carried out using the *snpReady* package in R statistical software (Version 4.5.1) [32]. This included filtering markers with minor allele frequency (MAF) < 0.05 and a call rate < 0.95. Imputation of missing markers was conducted using the *wright* method. Similarly, the *snpReady* package was used to estimate overall diversity estimates; observed heterozygosity (Ho), expected heterozygosity (He)/gene diversity (GD), polymorphic information content (PIC), and FIS were computed using the *snpReady* package [33]. Mutation types, transversion (Tv) and transition (Ts) were also computed. Pairwise fixation indices (FSTs), intra-cluster and inter-cluster diversity statistics were determined using the *dartR* package [34].

### 2.4. Population Structure Analysis

The population structure of the panel was inferred with the sparse non-negative matrix factorization (*snmf*) model implemented via the landscape and ecological associations (LEA) package in R [35]. The optimal number (K) of ancestral populations from the snmf model was determined using a cross-entropy criterion, with K varying from 1 to 10 and 10 independent runs per K. The optimal K was selected as the value that minimized the cross-entropy criterion [36]. Principal component analysis performed within LEA was used to confirm the number of genetic clusters.

### 2.5. Analysis of Molecular Variance

Analysis of molecular variance (AMOVA) was carried out to assess the genetic differentiation using the *poppr* package [37].

### 2.6. Hierarchical Clustering and Discriminant Analysis of Principal Components (DAPC)

To explore the genetic relationships among the accessions, discriminant analysis of principal components and cluster analysis were conducted. In the DAPC, the 10 best PCs were retained via stratified cross-validation. Agglomerative hierarchical clustering was performed using Ward’s minimum-variance method and Nei’s distance (Ward, D). The clusters were visualized using a dendrogram in R [38]. Discriminant analysis of principal components (DAPC) was carried out using the *adegenet* package [39].

## 3. Results

### 3.1. SNP Marker Characterization

**Figure 1:**
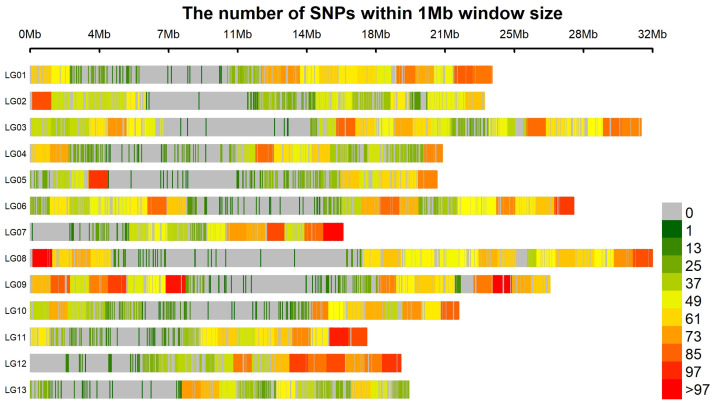
Distribution of SNP markers across sesame across 13 linkage groups within 1 Mb windows.

**Figure 2:**
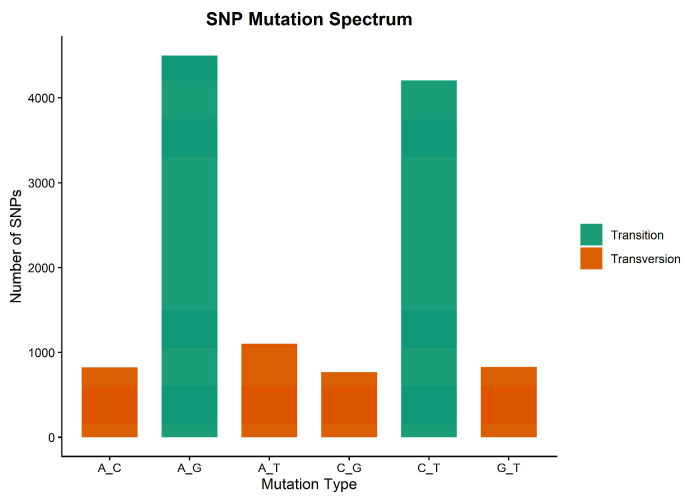
SNP mutation spectrum identified among the 11,502 SNPs used in the analysis of 109 sesame accessions.

A total of 14,763 DArTseq SNPs were generated, of which 12,230 were aligned to the *S. indicum* genome (genome assembly: ASM2616843v1; https://www.ncbi.nlm.nih.gov/datasets/genome/GCA_026168435.1/, accessed on 28 December 2025) and the remaining were unknown or scaffolds (Table 1, Figure 1). Filtering for MAF < 0.05 and a call rate < 0.95 yielded 11,502 high-quality SNPs for this study. The aligned markers were distributed across the 13 chromosomes with an average of 941 SNPs/chromosome. LG3 had the highest number of SNPs (1356), while LG13 had the lowest 596) (Table 1). The average SNP density was 40.4 SNPs/Mbp across the chromosomes, ranging from 30.56 SNPs/Mbp on LG13 to 47.49 SNPs/Mbp on LG12. The transition-to-transversion (Ts/Tv) ratio was 2.47, with transitions accounting for 71.2% (8706) of the total mutations and transversions accounting for 28.8% (3524) (Figure 2).

### 3.2. Genetic Diversity

The MAF of the 11,502 SNP markers ranged from 0.05 to 0.5 with a mean of 0.21, while the PIC ranged between 0.09 and 0.38 with a mean of 0.25 (Table 2). The mean genetic diversity (GD/He) was 0.30, and the observed heterozygosity was also 0.30. Within-cluster genetic diversity varied across the eight subpopulations, with higher observed heterozygosity than expected heterozygosity (Table 3). Cluster 6 (*n* = 29) had the highest number of polymorphic loci (10,161), followed by cluster 8 (*n* = 15, 9800 loci) and cluster 1 (*n* = 30, 9142 loci). Cluster 7 (*n* = 3) had the fewest polymorphic loci (6026). All subpopulations had negative inbreeding coefficients (FIS ranging from −0.28 to −0.36), while the whole panel had an FIS of 0.01.

### 3.3. Population Structure and Genetic Relationships

**Figure 3:**
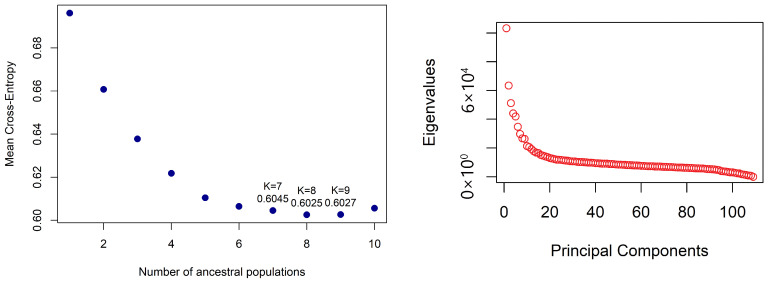
Cross-entropy and PCA scree plot-based population structure based on 11,502 sesame SNPs.

**Figure 4:**
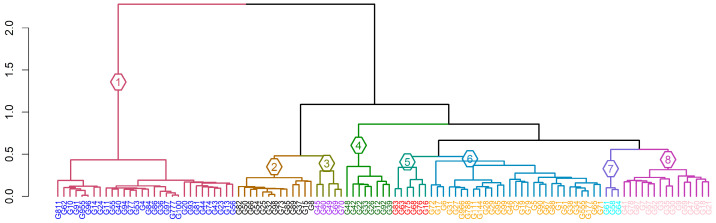
Dendrogram showing the 8 subpopulations in 109 sesame accessions based on 11,502 SNPs, with the numbers and colors representing each subpopulation.

The panel was grouped into eight subpopulations (K = 8) using the snmf model, yielding the lowest entropy (0.6025) (Figure 3). The hierarchical dendrogram grouped the genotypes into eight clusters (Figure 4). This result was confirmed by the PCA scree plot, which also started to plateau (forming an elbow) beginning at the eighth PC. The largest cluster, cluster 1, comprised 30 accessions, followed by cluster 6 (29 accessions). Cluster 7 was the smallest, with three accessions.

### 3.4. Discriminant Analysis of Principal Components (DAPC) and Admixture Analysis

**Figure 5:**
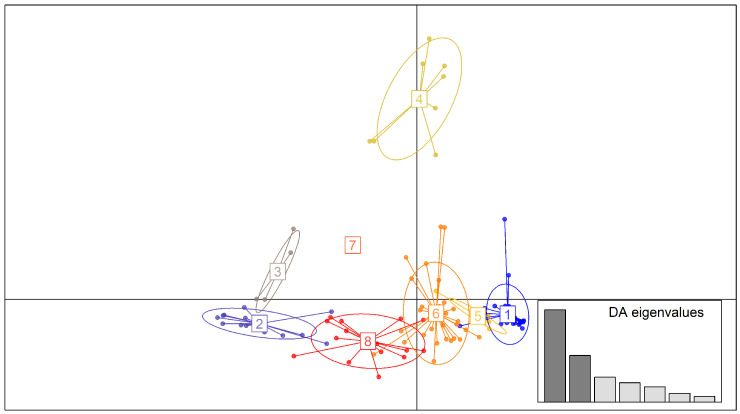
DAPC scatterplot of 8 subpopulations of 109 sesame accessions based on 11,502 SNPs, with each color representing a subpopulation.

**Figure 6:**
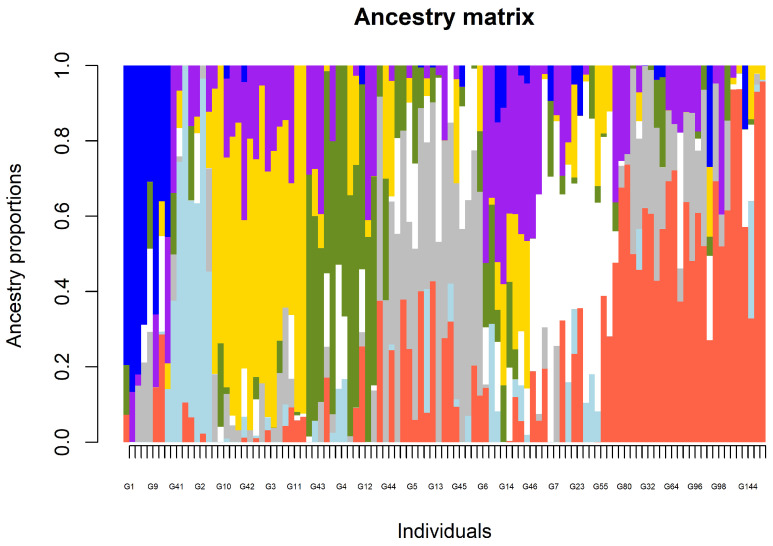
Genetic admixture proportions across 8 subpopulations of 109 sesame accessions based on 11,502 SNPs (The x-axis represents the accessions/individuals, while the colors represent each subpopulation).

Discriminant analysis of principal components (DAPC) visualized the genetic relationships of the accessions (Figure 5). The first two principal components captured the majority of the between-group variance. Clusters 2 and 3 were distinct from clusters 1, 5, and 6. Cluster 8 partially overlapped with cluster 6. Cluster 4 was clearly differentiated with minimal overlap with other clusters. Clusters 1, 5, and 6 also showed partial overlap; cluster 7 was the most differentiated, with minimal internal variance. The ancestry matrix (Figure 6) revealed substantial admixture levels across the panel. Few individuals showed pure assignment to a single ancestral lineage.

### 3.5. AMOVA

The analysis of molecular variance (AMOVA) partitioned the total genetic variation, revealing that the majority of the genetic variation (85.85%) was within the subpopulations, while 14.15% was among the subpopulations (Table 4). Moreover, pairwise analysis (FST) (Table 5) values ranged from low to moderate, i.e., 0.02 (subpopulation 8 and 6, subpopulation 8 and 5) to 0.1 (subpopulation 7 and 1). Subpopulation 7 showed the highest genetic divergence (0.05–0.10).

## 4. Discussion

Genetic diversity is essential for conservation and for the efficient use of germplasm in breeding programs [23]. Therefore, understanding the extent of genetic diversity and population structure provides useful information for strategic breeding and conservation efforts. Genetic characterization of sesame has been conducted using various molecular markers; however, genomic resources remain limited. This study investigated the extent of genetic diversity and population structure in 109 sesame accessions from Mozambique using genome-wide DArTseq single-nucleotide polymorphism (SNP) markers.

### 4.1. SNP Marker Coverages and Polymorphism

The 11,502 high-quality DArTseq SNPs generated in this study provided genome-wide coverage for investigating and characterizing the genetic diversity of this panel. The SNPs were well distributed across the 13 chromosomes; however, density varied within chromosomes. High-density regions on LG12 and LG9 indicate high recombination rates, whereas low-density regions on LG13 suggest lower polymorphism, possibly due to suppressed recombination at centromeres [40]. Similar non-uniform distributions have been reported in sesame [21,41]. Substitution mutation types affect polymorphism rates and are grouped as transition and transversion mutations. The predominance of transitions over transversions (Figure 2) could be attributed to the purine-pyrimidine stability of transitions during natural selection [42]. Similar dominance has been reported in *Brassica juncea* L. [43] and *Camelina sativa* L. [44].

### 4.2. Genetic Diversity of 109 Sesame Accessions

The overall genetic diversity (He = 0.30, Ho = 0.30, MAF = 0.21) in this study indicates a moderately broad genetic base among the 109 accessions. This agrees with earlier studies reporting He = 0.3 in 41 sesame accessions [21] and He = 0.332 in 501 sesame accessions [22], suggesting that sesame harbors substantial diversity. Polymorphism information content (PIC) estimates marker polymorphism and marker informativeness [45]. The mean PIC value of 0.25 indicates that the markers were moderately informative [46]. Previous studies in sesame have reported values ranging from 0.12 to 0.28 using SNPs and from 0.35 to 0.63 using SSRs [21,22,23,47,48]. This can be attributed to population origin and marker type, for example, because SNPs are biallelic and have a lower maximum PIC, whereas SSRs are multiallelic and have a higher PIC. Further, the chromosomal regions under selective pressure may alter allele frequency distributions and the number of alleles [49].

Population structure analysis provides insight into the genetic relatedness of individuals and is therefore useful for identifying diverse parents for hybridization. The panel in this study was grouped into eight subpopulations (K = 8) despite extensive admixture, suggesting that although the panel shares a common ancestral history, it still contains distinct subpopulations. Using the cross-entropy criterion in our structure analysis, K = 8 reached the absolute lowest cross-entropy value across K = 1–10 with 10 repetitions per K (Figure 3). Following the standard protocol for the *snmf* algorithm, the K value that minimizes this criterion (i.e., the lowest cross-entropy) is typically chosen as the most supported model under the sNMF framework for representing the population’s true genetic structure. This was further complemented by PCA implemented in the lfmm model. Previous studies in sesame have reported four [23], three [50,51,52], and two [21,53] subpopulations. The varying number of subpopulations may be attributed to the origin of accessions, population size, and the type of molecular marker used. The high admixture levels observed indicate historical seed-exchange practices, both locally and internationally, regeneration practices, and potential outcrossing in sesame [54]. Our findings align with earlier studies that reported varying degrees of admixture. For example, [51] using STRUCTURE and a membership threshold of <0.50 identified 7 accessions out of 95 as admixed, and [55] using a membership threshold of <0.60 identified 35 genotypes out of 129 as admixed.

All eight subpopulations showed negative FIS values (−0.28 to −0.36), indicating a systematic excess of heterozygotes, which is unlikely in self-pollinated crops. This pattern can be attributed to a combination of factors. First, the residual heterozygosity of landraces in the panel. Although sesame is predominantly self-pollinated, occasional outcrossing occurs at varying rates. For example, [47] reported outcrossing rates of approximately 45% and 46.4% in Ethiopian sesame landraces and cultivars, respectively, suggesting that the material consisted of line mixtures and segregants from outcrossing events. Under mixed-mating conditions, loci under balance or weak selection could remain unfixed for many generations. Second, admixture, as seen in the admixture analysis (Figure 6), suggests gene flow among lineages. Accessions of mixed ancestry may have contributed to heterozygosity. Third, scoring paralog loci as heterozygous SNPs is a limitation of reduced-representation platforms. According to [56], loci with Ho substantially exceeding Hardy–Weinberg equilibrium (HWE) expectation may represent collapsed paralogs. At the population level, Ho was equal to He; however, within each of the eight subpopulations, Ho was greater than He. Within subpopulations, heterozygous landrace genotypes may have inflated the Ho. However, at the population level, pooling all 109 diverse samples increased the overall allelic richness (reverse Wahlund effect). This inflated He to match Ho, creating the appearance of balance at the broader scale while masking the heterozygote excess within subpopulations. These findings collectively reflect the management of the panel and the complex domestication history of sesame, where human migration and trade facilitated germplasm exchange across geographical boundaries [57].

The DAPC supported the genetic relationships of the accessions with the first two axes explaining most of the variation. The DAPC partitioned the accessions in the panel into eight clusters, confirming the presence of distinct clusters. Overlapping clusters 1, 5, 6, and 8 in the DAPC supported the admixture patterns observed among the accessions, while differentiated cluster 7 suggested single-ancestry lineages consistent with the near-pure ancestry in the admixture plot. Cluster analysis further clearly separated the accessions into distinct clades, confirming the genetic diversity revealed by the SNP markers. At the same time, the assignment of accessions across the branches reflected the admixture patterns shaping the panel’s genetic structure. Cluster 1 was separated from other clusters by the largest distance, while clusters 2 through 8 were broadly complex. Clusters 7 and 8 revealed moderate similarity. The clustering pattern was consistent with genetic exchange or shared breeding history.

The higher variance within subpopulations (85.85%) than among subpopulations (14.15%) suggests that the accessions within subpopulations harbor a broad genetic base, providing a wide range of phenotypic traits useful for targeted selection. Consistent with our findings, previous studies on sesame have reported substantial genetic diversity within populations. Analysis by [58] using SSR markers reported 92% of variation within populations, while ref. [55,59] identified similar levels of genetic variation of 85.84% and 87.1%, respectively.

Further, the pairwise fixation indices (FSTs) quantified the differentiation between the subpopulations. Pairwise FST values < 0.05 indicate low differentiation, while values between 0.05 and 0.15 suggest moderate differentiation [60]. Our findings revealed that the subpopulations were slightly to moderately differentiated (Table 5). For example, a low fixation index (0.02) between subpopulations 5 and 8 suggests that the accessions in those subpopulations are genetically similar. This confirms the overlap observed in the DAPC. However, the moderate differentiation between subpopulations 1 and 7 (FST = 0.1) suggests that the subpopulations are genetically distinct. Accessions from those divergent subpopulations may be useful for exploring transgressive segregation for key agronomic traits.

## 5. Conclusions

The present study provided a genome-wide assessment of genetic diversity and population structure of a diverse panel of 109 sesame accessions from Mozambique using DArTseq SNP markers. The panel exhibited moderate overall genetic diversity (He = 0.30, MAF = 0.21), which is useful for potential breeding programs. Eight subpopulations were identified, indicating detectable genetic structure within the Mozambican germplasm. The high admixture levels indicate that while the accessions can form distinct clusters, they share an evolutionary history likely facilitated by historical germplasm exchange or localized gene flow. The excess heterozygosity within subpopulations likely reflects residual heterozygosity in sesame landraces, admixture effects, and potential scoring of paralogs as heterozygous SNPs. The high within-subpopulation molecular variance (85.85%) suggests useful selectable variation exists within as well as among subpopulations. The pairwise fixation indices identified moderately differentiated accessions in subpopulations 7 and 1 as potential candidates for hybridization. Overall, these findings provide a foundation for informed parental selection in future sesame breeding programs in Mozambique.

## Figures and Tables

**Table 1:** Genomic distribution of 14,763 DArTseq SNPs across sesame chromosomes.

Chromosome	Length (Mbp)	No. of SNPs	Marker Distance (Kbp)	SNP_per_Mbp
LG1	23.75	1044	22.75	43.96
LG2	23.37	815	28.67	34.87
LG3	31.44	1356	23.19	43.13
LG4	21.23	797	26.64	37.54
LG5	20.96	657	31.9	31.35
LG6	27.99	1220	22.94	43.59
LG7	16.12	716	22.51	44.42
LG8	31.99	1252	25.55	39.14
LG9	26.74	1249	21.41	46.71
LG10	22.21	825	26.92	37.15
LG11	17.33	796	21.77	45.93
LG12	19.1	907	21.06	47.49
LG13	19.5	596	32.72	30.56
Scaffolds	-	49	-	-
Unknown	-	2484	-	-
Total	301.73	14,763	328.03	525.84
Average	-	941	25.2	40.4

Chromosome names and length according to the genome assembly, https://www.ncbi.nlm.nih.gov/datasets/genome/GCA_026168435.1/ (accessed on 17 March 2026).

**Table 2:** Genetic diversity parameters of the sesame population based on 11,502 SNPs.

	Mean	Lower	Upper
He/GD	0.3	0.1	0.5
PIC	0.25	0.09	0.38
MAF	0.21	0.05	0.5
Ho	0.3	0.08	0.55
FIS	0.01	−0.83	0.72

**Table 3:** Subpopulation diversity estimates.

Pop	n.Ind	polyLoc	monoLoc	Ho	He	FIS
6	29	10,161	1341	0.37	0.26	−0.3
2	13	8408	3094	0.37	0.24	−0.36
1	30	9142	2360	0.37	0.24	−0.35
8	15	9800	1702	0.38	0.26	−0.28
4	8	8794	2708	0.37	0.25	−0.29
3	5	7704	3798	0.38	0.24	−0.31
7	3	6026	5476	0.37	0.22	−0.35
5	6	8189	3313	0.38	0.25	−0.3

**Table 4:** Analysis of molecular variance between and within sesame accessions.

Source of Variance	Df	Sum Sq	Mean Sq	% Variance	*p*-Value
Between samples	7	90,349.87	12,907.13	14.15	
Within samples	101	423,713.39	4195.182	85.85	
Total	108	514,063.27	4759.845	100	<0.001

**Table 5:** Fixation indices across the 8 sesame subpopulations.

	Pop 6	Pop 2	Pop 1	Pop 8	Pop 4	Pop 3	Pop 7
Pop 2	0.04	NA	NA	NA	NA	NA	NA
Pop 1	0.03	0.08	NA	NA	NA	NA	NA
Pop 8	0.02	0.03	0.05	NA	NA	NA	NA
Pop 4	0.03	0.07	0.04	0.04	NA	NA	NA
Pop 3	0.04	0.04	0.07	0.03	0.06	NA	NA
Pop 7	0.06	0.07	0.1	0.05	0.08	0.06	NA
Pop 5	0.03	0.05	0.05	0.02	0.05	0.04	0.07

NA represents Not Applicable.

## Data Availability

The data used in this study are available in the article and Supplementary Materials. Further inquiries should be directed to the corresponding author.

## Supplementary Materials

The following supporting information can be downloaded at https://www.mdpi.com/article/10.3390/genes17050528/s1, Table S1: List of Sesame Genotypes and their identified clusters.

## Abbreviations

The following abbreviations are used in this manuscript:

RAPD	Random amplified polymorphic DNA
SSRs	Simple sequence repeats
ISSRs	Inter-simple sequence repeats
AFLPs	Amplified fragment length polymorphisms
SRAPs	Sequence-related amplified polymorphisms
SNPs	Single-nucleotide polymorphisms
DArTseq	Diversity array technology sequencing
USDA	US Arid Land Agricultural Research Center
WGS	Whole-genome resequencing
MAF	Minor allele frequency
He	Expected heterozygosity
GD	Genetic diversity
Ho	Observed heterozygosity
FST	Fixation index
FIS	Coefficient of inbreeding
AMOVA	Analysis of molecular variance
DAPC	Discriminant analysis of principal components
Va	Additive variance
Vd	Dominant variance
Ne	Effective population size
Pop	Population
n.ind	Number of individuals
Polyloc	Polymorphic loci
Monoloc	Monomorphic loci
A/G	Adenine/Guanine
C/T	Cytosine/Thymine
PIC	Polymorphic information content
Df	Degree of freedom
NA	Not Applicable
