# Supplementary material for: Genetic Characterization and Population Structure of Mozambique’s Sesame (Sesamum indicum L.) Accessions Using DArTseq-Derived SNP Markers
Source: Genes (Basel). 2026 Apr 29;17(5):528. doi: 10.3390/genes17050528 (PMC13205522; doi:10.3390/genes17050528)
Supplement: Supplementary file 1 [file genes-17-00528-s001.zip › genes-4273168-supplementary.pdf]

Supplementary Table S1: List of Sesame Genotypes and their identified clusters.

No.	Genotype	Cluster
1	G4	1
2	G10	1
3	G11	1
4	G13	1
5	G14	1
6	G20	1
7	G23	1
8	G24	1
9	G36	1
10	G40	1
11	G43	1
12	G44	1
13	G53	1
14	G55	1
15	G56	1
16	G62	1
17	G74	1
18	G77	1
19	G81	1
20	G84	1
21	G86	1
22	G91	1
23	G93	1
24	G94	1
25	G96	1
26	G97	1
27	G100	1
28	G777	1
29	G811	1
30	G955	1
31	G8	2
32	G15	2
33	G25	2
34	G32	2
35	G37	2
36	G50	2
37	G54	2
38	G66	2

39	G73	2
40	G82	2
41	G85	2
42	G89	2
43	G98	2
44	G45	3
45	G49	3
46	G69	3
47	G70	3
48	G80	3
49	G12	4
50	G22	4
51	G26	4
52	G33	4
53	G39	4
54	G42	4
55	G48	4
56	G92	4
57	G16	5
58	G63	5
59	G68	5
60	G75	5
61	G76	5
62	G83	5
63	G1	6
64	G2	6
65	G5	6
66	G6	6
67	G17	6
68	G19	6
69	G27	6
70	G28	6
71	G29	6
72	G31	6
73	G34	6
74	G38	6
75	G46	6
76	G51	6
77	G65	6
78	G67	6
79	G71	6
80	G72	6

81	G79	6
82	G88	6
83	G90	6
84	G95	6
85	G99	6
86	G111	6
87	G144	6
88	G177	6
89	G555	6
90	G788	6
91	G966	6
92	G58	7
93	G61	7
94	G64	7
95	G3	8
96	G7	8
97	G9	8
98	G18	8
99	G21	8
100	G30	8
101	G35	8
102	G41	8
103	G47	8
104	G52	8
105	G57	8
106	G59	8
107	G60	8
108	G78	8
109	G87	8
